# A Country-Wide Study of Spoligotype and Drug Resistance Characteristics of *Mycobacterium tuberculosis* Isolates from Children in China

**DOI:** 10.1371/journal.pone.0084315

**Published:** 2013-12-30

**Authors:** Weiwei Jiao, Zhiguang Liu, Rui Han, Xiuqin Zhao, Fang Dong, Haiyan Dong, Hairong Huang, Jianling Tian, Qinjing Li, Lulu Lian, Qingqin Yin, Wenqi Song, Kanglin Wan, A-dong Shen

**Affiliations:** 1 Key Laboratory of Major Diseases in Children and National Key Discipline of Pediatrics (Capital Medical University), Ministry of Education, Beijing Pediatric Research Institute, Beijing Children’s Hospital, Capital Medical University, Beijing, China; 2 National Institute for Communicable Disease Control and Prevention, Chinese Center for Disease Control and Prevention, State Key Laboratory for Infectious Disease Prevention and Control, Beijing, China; 3 Collaborative Innovation Center for Diagnosis and Treatment of Infectious Diseases, Hangzhou, Zhejiang, China; 4 National Tuberculosis Clinical Laboratory, Beijing Chest Hospital, Capital Medical University, Beijing, China; St. Petersburg Pasteur Institute, Russian Federation

## Abstract

**Background:**

Tuberculosis (TB) is still a big threat to human health, especially in children. However, an isolation of *Mycobacterium tuberculosis* culture from pediatric cases remains a challenge. In order to provide some scientific basis for children TB control, we investigated the genotyping and drug resistance characteristics of *M. tuberculosis* isolates from pediatric cases in China.

**Methodology/Principal Findings:**

In this study, a total of 440 strains including 90 from children (<15 years), 159 from adolescents (15–18 years) and 191 from adults (>18 years) isolated in 25 provinces across China were subjected to spoligotyping and drug susceptibility testing. As a result, Beijing family strains were shown to remain predominant in China (85.6%, 81.1% and 75.4% in three above groups, respectively), especially among new children cases (91.0% vs. 69.6% in previously treated cases, P = 0.03). The prevalence of the Beijing genotype isolates was higher in northern and central China in the total collection (85.1% in northern and 83.9% in central vs. 61.6% in southern China, P<0.001) and a similar trend was seen in all three age groups (P = 0.708, <0.001 and 0.025, respectively). In adolescents, the frequencies of isoniazid (INH)-resistant and ethambutol (EMB)-resistant isolates were significantly higher among Beijing strains compared to non-Beijing genotype strains (P = 0.028 for INH and P = 0.027 for EMB). Furthermore, strong association was observed between resistance to rifampicine (RIF), streptomycin (STR) and multidrug resistance (MDR) among Beijing compared to non-Beijing strains in previously treated cases of children (P = 0.01, 0.01 and 0.025, respectively).

**Conclusion/Significance:**

Beijing family was more prevalent in northern and central China compared to southern China and these strains were predominant in all age groups. The genetic diversity of *M. tuberculosis* isolates from children was similar to that found in adolescents and adults. Beijing genotype was associated with RIF, STR and MDR resistance in previously treated children.

## Introduction

Tuberculosis (TB), caused by *Mycobacterium tuberculosis* (*M. tuberculosis*), still remains a major threat to human health. TB among children is especially important to public health workers since it is an indicator of recent transmission in the community. However, data on pediatric TB is limited and often underestimated because of diagnostic challenges. According to WHO tuberculosis report in 2012, there were estimated 0.5 million TB cases and 64,000 deaths among children in 2011 [1]. In the 37 countries providing multidrug-resistant tuberculosis [MDR-TB, characterized by resistance to at least isoniazid (INH) and rifampicin (RIF)] data including children, children represented 1–13% of total enrolments [1]. This was also the first time to include estimates of TB burden among children in the global tuberculosis report.

China is one of the high burden countries, ranking the second of total TB patients globally. However the most recent available data of TB in children came from the fourth national TB survey conducted in 2000. It was estimated that the prevalence of TB among 0–14 years old children was 9%, and there were 266,000 children cases with active pulmonary TB, making a serious threat to children health in China [2]. Therefore, more attention should be paid to *M. tuberculosis* strains isolated from children, which can provide the useful information for effective TB control.

The molecular typing methods have become the useful tool in the control of TB, which help to indicate possible epidemiological links between TB patients, detect the outbreaks and laboratory cross-contamination. Based on the DNA polymorphisms of *M. tuberculosis* genome, a number of genotyping methods have been developed and widely used in *M. tuberculosis* molecular typing. Spacer oligonucleotide typing (spoligotyping) is one of the easy-to-do and standardized typing methods [3] although its discriminatory power is limited. However, spoligotyping has a continuing updated international spoligotype database [4], and also, it has long been considered the gold standard for identifying strains belonging to the Beijing family [5].

In this study, we investigated *M. tuberculosis* strains isolated from Chinese children (less than 15 years), adolescents (15–18 years) and adults (more than 18 years) using spoligotyping and drug susceptibility testing. The obtained results on genotypes and drug resistance in three age groups were compared to data on treatment status and patients’ origin.

## Materials and Methods

### Ethics Statement

The research was approved by the Ethics Committee of Beijing Children’s Hospital, Capital Medical University and National Institute for Communicable Disease Control and Prevention, Chinese Center for Disease Control and Prevention. The patients and parents/guardians on behalf of the children and adolescents included in this study were given an information consent form and they all signed to participate in the study.

### *M. tuberculosis* Isolates

*M. tuberculosis* isolates were selected from the *M. tuberculosis* strain bank of Chinese Center for Disease Control and Prevention. The clinical strains in the bank were isolated from body fluid samples (sputum, bronchoalveolar lavage fluid, cerebrospinal fluid, pleural effusion, blood, or gastric juice) of the patients confirmed with TB in institutes for TB control and cure, as well as TB hospitals distributed in the following 25 provinces, municipalities and autonomous regions across China: Anhui, Beijing, Chongqing, Fujian, Gansu, Guangxi, Guizhou, Hebei, Henan, Hunan, Hubei, Heilongjiang, Inner Mongolia, Jilin, Jiangsu, Jiangxi, Liaoning, Qinghai, Shandong, Shaanxi, Shanxi, Sichuan, Tianjin, Xinjiang, and Xizang (Tibet). All the strains in the bank from patients less than 18 years old were included in this study, and the other group of strains from patients more than 18 years old was selected using random number table matched by region and isolation time. The basic information of eligible patients was also collected. Patients with new cases were those who had tuberculosis that had never been treated with anti-tuberculosis drugs or that had been treated for less than 1 month. Patients with previously treated cases were those who had been treated for tuberculosis for 1 month or longer.

### Drug Susceptibility Test

The chosen strains were recovered on Loewenstein-Jensen medium for 4 weeks at 37°C. Drug susceptibility testing to four first-line anti-TB drugs [INH, RIF, ethambutol (EMB) and streptomycin (STR)] was performed by proportion method as recommended by WHO [6]. The concentrations of different drugs in the media were as following: INH 0.2 µg/mL, RIF 40 µg/mL, EMB 2 µg/mL, and STR 4 µg/mL. The strain was considered resistant to specific drug when the growth rate was more than 1% compared to the control.

### Spoligotyping

Total chromosomal DNA of strains was isolated following recommended method described by van Embden et al. [7]. Spoligotyping was performed as described by Kamerbeek et al. [3]. In short, the PCR amplified biotin-labeled DR locus was hybridized against membrane with immobilized 43 different DR spacers using Miniblotter MN45 apparatus (Immunetics, Cambridge, MA, USA). Resulting hybridization signals were revealed by chemiluminescence and visualized as profiles of discrete dots. The spoligoprofiles were entered into Excel spreadsheets and compared with SITVITWEB international spoligotype database [4].

### Statistical Analysis

Tests for association between various factors (geographical distribution, drug resistance, new cases or previously treated cases) and genotypes were performed using the chi-square test with IBM SPSS Statistics 19. The level of significance was set at P<0.05.

## Results

### Study Population

A total of 440 isolates were selected for this study, including 90 from children, 159 from adolescents, and 191 from adults. The male patients were prevalent in all three age groups and constituted 55.6% (50/90), 52.2% (83/159) and 63.9% (122/191), respectively. The average age of patients in each group was 7.3 years, 16.9 years and 40.8 years. Newly diagnosed cases constitute 308 (70.0%) individuals in our collection, and accounted for 74.4% (67/90), 69.8% (111/159) and 68.1% (130/191) among patients from children, adolescents and adults, respectively. All the patients enrolled in this study were proven to be unlinked based on conventional epidemiological investigation.

### Distribution of Spoligotypes in Different Groups

In the 440 typed isolates, a total of 66 spoligotypes were identified (Table 1). Of them, 47 spoligotypes were present in the SITVITWEB database, while other 15 spoligotypes were not found in SITVITWEB and were named as New 1 to New 15 (Table 1). Family assignment revealed that 350 isolates (79.5%) belonged to the Beijing family, followed by ill-defined T superfamily (12.7%, 56/440) and Haarlem family (2.0%, 9/440). In addition, strains from other families, such as CAS1-Delhi family, LAM9 family, S family, Manu_ancestor family, MANU2 family, were also found in this study although at very low rate (0.2% for all families except for 0.4% for LAM9 family).

**Table 1:** Distribution of spoligotype families in different groups.

SIT*	Family	Spoligoprofile	All	Children	Adolescents	Adults
			n (%)	n (%)	n (%)	n (%)
1	Beijing	□□□□□□□□□□□□□□□□□□□□□□□□□□□□□□□□□□▪▪▪▪▪▪▪▪▪	322 (73.2)	71 (78.9)	118 (74.2)	133 (69.6)
190	Beijing	□□□□□□□□□□□□□□□□□□□□□□□□□□□□□□□□□□▪▪▪▪▪□▪▪▪	11 (2.5)	3 (3.3)	4 (2.5)	4 (2.1)
250	Beijing	□□□□□□□□□□□□□□□□□□□□□□□□□□□□□□□□□□□□□▪▪▪▪▪▪	1 (0.2)	0 (0)	1 (0.6)	0 (0)
260	Beijing	□□□□□□□□□□□□□□□□□□□□□□□□□□□□□□□□□□▪▪□□▪▪▪▪▪	1 (0.2)	0 (0)	1 (0.6)	0 (0)
265	Beijing	□□□□□□□□□□□□□□□□□□□□□□□□□□□□□□□□□□▪▪□▪▪▪▪▪▪	1 (0.2)	0 (0)	1 (0.6)	0 (0)
269	Beijing	□□□□□□□□□□□□□□□□□□□□□□□□□□□□□□□□□□□□▪▪▪▪▪▪▪	1 (0.2)	1 (1.1)	0 (0)	0 (0)
621	Beijing	□□□□□□□□□□□□□□□□□□□□□□□□□□□□□□□□□□▪□▪▪▪▪▪▪▪	1 (0.2)	1 (1.1)	0 (0)	0 (0)
632	Beijing	□□□□□□□□□□□□□□□□□□□□□□□□□□□□□□□□□□▪▪▪□▪▪▪▪▪	2 (0.4)	0 (0)	1 (0.6)	1 (0.5)
1162	Beijing	□□□□□□□□□□□□□□□□□□□□□□□□□□□□□□□□□□▪□□□▪▪▪▪▪	2 (0.4)	0 (0)	0 (0)	2 (1.0)
1311	Beijing	□□□□□□□□□□□□□□□□□□□□□□□□□□□□□□□□□□▪▪▪▪▪□□□□	1 (0.2)	0 (0)	1 (0.6)	0 (0)
1364	Beijing	□□□□□□□□□□□□□□□□□□□□□□□□□□□□□□□□□□▪▪▪□□▪▪▪▪	1 (0.2)	0 (0)	0 (0)	1 (0.5)
1674	Beijing	□□□□□□□□□□□□□□□□□□□□□□□□□□□□□□□□□□▪▪▪▪▪▪▪□▪	1 (0.2)	0 (0)	0 (0)	1 (0.5)
2101	Beijing	□□□□□□□□□□□□□□□□□□□□□□□□□□□□□□□□□□▪▪□□□□□□□	3 (0.7)	0 (0)	2 (1.3)	1 (0.5)
2413	Beijing	□□□□□□□□□□□□□□□□□□□□□□□□□□□□□□□□□□▪▪▪▪▪▪▪□□	1 (0.2)	1 (1.1)	0 (0)	0 (0)
2610	Beijing	□□□□□□□□□□□□□□□□□□□□□□□□□□□□□□□□□□▪▪▪▪▪▪▪▪□	1 (0.2)	0 (0)	0 (0)	1 (0.5)
53	T1	▪▪▪▪▪▪▪▪▪▪▪▪▪▪▪▪▪▪▪▪▪▪▪▪▪▪▪▪▪▪▪▪□□□□▪▪▪▪▪▪▪	25 (5.7)	4 (4.4)	5 (3.1)	16 (8.4)
167	T1	▪▪▪▪▪▪▪▪▪▪▪▪▪▪▪▪▪▪▪▪▪▪▪▪▪▪▪▪▪□▪▪□□□□▪▪▪▪▪▪▪	1 (0.2)	0 (0)	1 (0.6)	0 (0)
242	T1	▪▪▪▪▪▪▪▪▪▪▪▪▪▪▪▪▪▪▪▪▪▪▪▪▪▪▪▪▪▪▪▪□□□□▪□□□▪▪▪	1 (0.2)	0 (0)	0 (0)	1 (0.5)
291	T1	▪▪▪▪▪▪▪▪▪▪▪▪▪▪▪▪▪▪▪▪□▪▪▪▪▪▪▪▪▪▪▪□□□□▪▪▪▪▪▪▪	1 (0.2)	0 (0)	1 (0.6)	0 (0)
334	T1	▪□▪▪▪▪▪▪▪▪▪▪▪▪▪▪▪▪▪▪▪▪▪▪▪▪▪▪▪▪▪▪□□□□▪▪▪▪▪▪▪	1 (0.2)	0 (0)	0 (0)	1 (0.5)
393	T1	▪▪▪▪▪▪▪▪▪▪▪▪▪□▪▪▪▪▪▪▪▪▪▪▪▪▪▪▪▪▪▪□□□□▪▪▪▪▪▪▪	1 (0.2)	0 (0)	0 (0)	1 (0.5)
462	T1	▪▪▪▪▪▪▪▪▪▪▪▪▪▪▪▪▪▪▪▪▪▪▪▪▪▪▪▪□▪▪▪□□□□▪▪▪▪▪▪▪	1 (0.2)	0 (0)	0 (0)	1 (0.5)
751	T1	□□□▪▪▪▪▪▪▪▪▪▪▪▪▪▪▪▪▪▪▪▪▪▪▪▪▪▪▪▪▪□□□□▪▪▪▪▪▪▪	1 (0.2)	0 (0)	1 (0.6)	0 (0)
834	T1	▪▪▪▪▪▪▪▪▪▪▪▪▪▪□▪▪▪▪▪▪▪▪▪▪▪▪▪▪▪▪□□□□□▪▪▪▪▪▪▪	1 (0.2)	0 (0)	1 (0.6)	0 (0)
913	T1	▪▪▪▪▪▪▪▪▪▪▪▪▪□□□▪▪▪▪▪▪▪▪▪▪▪▪▪▪▪▪□□□□▪▪▪▪▪▪▪	1 (0.2)	0 (0)	0 (0)	1 (0.5)
1688	T1	▪▪▪▪▪▪▪▪▪▪▪▪▪▪▪▪▪▪▪□□□□□□▪▪▪▪▪▪▪□□□□▪▪▪▪▪▪▪	1 (0.2)	0 (0)	0 (0)	1 (0.5)
2037	T1	▪▪▪▪▪▪▪▪▪▪▪▪▪▪▪▪▪▪▪▪▪▪▪▪▪□□□▪▪▪▪□□□□▪▪▪▪▪▪▪	1 (0.2)	0 (0)	1 (0.6)	0 (0)
Orphan	T1	▪▪▪▪▪▪▪▪▪▪▪▪▪▪▪▪▪▪▪▪▪▪▪▪▪▪▪▪▪□▪▪□□□□□□□▪▪▪▪	1 (0.2)	1 (1.1)	0 (0)	0 (0)
52	T2	▪▪▪▪▪▪▪▪▪▪▪▪▪▪▪▪▪▪▪▪▪▪▪▪▪▪▪▪▪▪▪▪□□□□▪▪▪□▪▪▪	9 (2.0)	1 (1.1)	4 (2.5)	4 (2.1)
Orphan	T2	▪▪▪▪▪▪▪▪▪▪▪▪▪▪▪▪▪▪▪▪▪▪▪▪▪▪▪▪□▪▪▪□□□□▪▪▪□▪▪▪	1 (0.2)	0 (0)	0 (0)	1 (0.5)
Orphan	T2	▪▪▪▪▪▪▪▪▪▪▪▪▪▪▪▪▪▪▪▪▪▪▪□▪▪▪▪▪▪▪▪□□□□▪▪▪□▪▪▪	2 (0.4)	0 (0)	0 (0)	2 (1.0)
37	T3	▪▪▪▪▪▪▪▪▪▪▪▪□▪▪▪▪▪▪▪▪▪▪▪▪▪▪▪▪▪▪▪□□□□▪▪▪▪▪▪▪	2 (0.4)	0 (0)	1 (0.6)	1 (0.5)
1547	T3	▪▪▪▪▪▪▪▪▪▪▪▪□▪□▪▪▪▪▪▪▪▪▪▪▪▪▪▪▪▪▪□□□□▪▪▪▪▪▪▪	1 (0.2)	1 (1.1)	0 (0)	0 (0)
Orphan	T3	▪□▪▪▪▪▪▪▪▪▪▪□▪▪▪▪▪▪▪▪▪▪▪▪▪▪▪▪▪▪▪□□□□▪▪▪▪▪▪▪	3 (0.7)	1 (1.1)	1 (0.6)	1 (0.5)
44	T5	▪▪▪▪▪▪▪▪▪▪▪▪▪▪▪▪▪▪▪▪▪▪□▪▪▪▪▪▪▪▪▪□□□□▪▪▪▪▪▪▪	1 (0.2)	0 (0)	1 (0.6)	0 (0)
47	H1	▪▪▪▪▪▪▪▪▪▪▪▪▪▪▪▪▪▪▪▪▪▪▪▪▪□□□□□□▪□□□□▪▪▪▪▪▪▪	1 (0.2)	0 (0)	0 (0)	1 (0.5)
49	H3	▪▪▪▪▪▪▪▪▪▪▪▪▪▪▪▪▪▪▪▪▪▪▪▪▪▪▪▪▪▪□▪□□□□▪▪▪□▪▪▪	1 (0.2)	0 (0)	1 (0.6)	0 (0)
50	H3	▪▪▪▪▪▪▪▪▪▪▪▪▪▪▪▪▪▪▪▪▪▪▪▪▪▪▪▪▪▪□▪□□□□▪▪▪▪▪▪▪	2 (0.4)	0 (0)	1 (0.6)	1 (0.5)
512	H3	▪▪▪▪▪▪▪▪▪▪▪▪▪▪▪▪▪▪▪▪▪□□□▪▪▪▪▪▪□▪□□□□▪▪▪▪▪▪▪	1 (0.2)	0 (0)	1 (0.6)	0 (0)
742	H3	▪▪▪▪▪▪▪▪▪▪▪▪▪▪▪▪▪▪▪▪▪▪▪▪□□□□□□□▪□□□□▪▪▪▪▪▪▪	1 (0.2)	0 (0)	0 (0)	1 (0.5)
2092	H3	▪▪▪▪▪▪▪▪▪▪▪▪□▪▪▪▪▪▪▪▪▪▪▪□□□□□□□▪□□□□▪▪▪▪▪▪▪	1 (0.2)	0 (0)	0 (0)	1 (0.5)
127	H4	▪□▪▪▪▪▪▪▪▪▪▪▪▪▪▪▪▪▪▪▪▪▪▪▪▪▪▪□□□▪□□□□▪▪▪▪▪▪▪	2 (0.4)	0 (0)	0 (0)	2 (1.0)
26	CAS1-Delhi	▪▪▪□□□□▪▪▪▪▪▪▪▪▪▪▪▪▪▪▪□□□□□□□□□□□□▪▪▪▪▪▪▪▪▪	1 (0.2)	0 (0)	1 (0.6)	0 (0)
803	LAM9	▪▪▪▪▪▪▪▪▪▪▪▪▪□□□□□□□□□□□▪▪▪▪▪▪▪▪□□□□▪▪▪▪▪▪▪	2 (0.4)	1 (1.1)	0 (0)	1 (0.5)
34	S	▪▪▪▪▪▪▪▪□□▪▪▪▪▪▪▪▪▪▪▪▪▪▪▪▪▪▪▪▪▪▪□□□□▪▪▪▪▪▪▪	1 (0.2)	0 (0)	1 (0.6)	0 (0)
523	Manu_ancestor	▪▪▪▪▪▪▪▪▪▪▪▪▪▪▪▪▪▪▪▪▪▪▪▪▪▪▪▪▪▪▪▪▪▪▪▪▪▪▪▪▪▪▪	1 (0.2)	0 (0)	0 (0)	1 (0.5)
54	MANU2	▪▪▪▪▪▪▪▪▪▪▪▪▪▪▪▪▪▪▪▪▪▪▪▪▪▪▪▪▪▪▪▪□□▪▪▪▪▪▪▪▪▪	1 (0.2)	0 (0)	0 (0)	1 (0.5)
602	unknown	▪▪▪▪▪▪▪▪▪▪▪▪▪▪▪▪▪▪▪▪▪▪▪▪□□□□□□□□□□□□▪▪▪▪▪▪▪	1 (0.2)	1 (1.1)	0 (0)	0 (0)
623	unknown	▪▪▪▪▪▪▪▪▪▪▪▪▪▪▪▪▪▪▪▪▪▪□▪▪▪▪▪▪▪▪▪▪▪▪▪▪▪▪▪▪▪▪	1 (0.2)	0 (0)	1 (0.6)	0 (0)
1410	unknown	▪▪▪▪▪▪▪▪▪▪▪▪▪▪▪▪▪▪▪▪▪□□□□□□□□□□□□□□□□□□□□□□	1 (0.2)	0 (0)	0 (0)	1 (0.5)
2731	unknown	▪▪▪▪▪▪▪▪▪▪▪▪▪▪▪▪▪▪▪▪▪▪▪▪▪▪▪▪▪▪▪▪□▪▪▪▪▪▪▪▪▪▪	1 (0.2)	0 (0)	1 (0.6)	0 (0)
New1	unknown	▪▪▪▪▪▪▪▪▪▪▪▪▪▪▪▪▪▪▪▪▪▪▪▪▪▪□□□□▪▪□□□□▪▪▪▪▪▪▪	1 (0.2)	0 (0)	1 (0.6)	0 (0)
New2	unknown	▪▪▪▪▪▪▪▪▪▪▪▪▪▪▪▪▪▪▪▪□▪▪▪□▪▪▪▪▪▪▪□□□□▪▪▪▪▪▪▪	1 (0.2)	0 (0)	0 (0)	1 (0.5)
New3	unknown	▪▪▪▪▪▪▪▪▪▪▪□▪□□□□□□□□□□□▪▪▪▪▪▪▪▪□□□□▪▪▪▪▪▪▪	1 (0.2)	1 (1.1)	0 (0)	0 (0)
New4	unknown	▪▪▪▪▪▪▪▪□□□□□□□□□□□▪▪▪▪▪▪▪▪▪▪▪▪▪□□□□▪▪▪▪▪▪▪	1 (0.2)	0 (0)	1 (0.6)	0 (0)
New5	unknown	▪▪▪▪▪□▪▪▪▪▪▪▪▪▪▪▪▪▪▪▪▪▪▪□□□□□□□▪□□□□▪▪▪▪▪▪▪	1 (0.2)	0 (0)	1 (0.6)	0 (0)
New6	unknown	▪▪▪▪▪□□□▪▪□▪▪▪▪▪▪▪▪▪▪▪▪▪▪▪▪▪▪▪▪▪□□□□▪▪▪□▪▪▪	1 (0.2)	0 (0)	0 (0)	1 (0.5)
New7	unknown	▪▪▪▪▪□□□□□□□▪▪▪▪▪▪▪▪▪▪□▪▪▪▪▪▪▪▪▪□□□□▪▪▪▪▪▪▪	1 (0.2)	0 (0)	1 (0.6)	0 (0)
New8	unknown	▪▪▪□□□□▪▪▪▪▪▪□□□□□□□□□□□□□□□□□□□□□▪▪□□▪▪▪▪▪	1 (0.2)	0 (0)	0 (0)	1 (0.5)
New9	unknown	▪▪□▪▪▪▪▪▪▪▪▪▪▪▪▪▪▪▪▪▪▪▪▪□▪▪▪▪▪▪▪□□□□▪▪▪□□□□	1 (0.2)	0 (0)	0 (0)	1 (0.5)
New10	unknown	▪▪□▪▪▪▪▪▪▪▪▪▪▪▪▪□▪▪▪□□□□▪▪▪▪▪▪▪▪□□▪▪▪▪▪▪▪▪▪	1 (0.2)	0 (0)	0 (0)	1 (0.5)
New11	unknown	▪□□□□□□□□□□□□□□□□□□□□□□□□□□□□□□□□□□□□□□□□▪▪	1 (0.2)	0 (0)	1 (0.6)	0 (0)
New12	unknown	□▪▪▪▪▪▪▪□▪▪▪▪▪▪▪▪▪▪▪▪▪▪▪▪▪▪▪▪▪▪▪□□□□▪▪▪□▪▪▪	1 (0.2)	1 (1.1)	0 (0)	0 (0)
New13	unknown	□□□□□□□□□□□□□□□□□□□□□□□□□□□□□□□□□□▪▪▪▪□□□▪▪	1 (0.2)	1 (1.1)	0 (0)	0 (0)
New14	unknown	□□□□□□□□□□□□□□□□□□□□□□□□□□□□□□□□□□▪▪□□□□□▪▪	1 (0.2)	0 (0)	0 (0)	1 (0.5)
New15	unknown	□□□□□□□□□□□□□□□□□□□□□□□□□□□□□□□□□□▪□▪▪▪▪▪▪□	1 (0.2)	0 (0)	1 (0.6)	0 (0)

SIT and family were defined by comparison with SITVITWEB; ‘new’ designates spoligotype not found in SITVITWEB.

Among 90 strains isolated from children, the Beijing family was predominant (85.6%, 77/90), followed by T family (8.9%, 8/90) and LAM family (1.1%, 1/90) (Table 1). According to the SITVITWEB database, 87 pediatric isolates were assigned to the known SITs, while three isolates presented new patterns (New3, New12 and New13). Seventy-one (78.9%) isolates showed a profile typical of the Beijing genotype (SIT1), and six had abridged Beijing-like spoligoprofiles (SIT190, SIT269, SIT621, SIT2413). The remaining 13 isolates were subdivided into 10 genotypes. The isolates from adolescents and adults were divided into five and seven clusters, respectively. The most prevalent family in the two groups was also the Beijing family (81.1% in adolescents, 75.4% in adults), with T family (10.7% in adolescents, 16.2% in adults) ranking the second (Table 1). There was no significant difference in the distribution of the Beijing family strains among the three age groups (P = 0.123).

### Geographical Distribution of Beijing Family Strains in Different Age Groups

According to the geographical division suggested by Wan et al. [8], the region of origin of strains were defined as (i) northern China (to the north of Yellow River, and Xizang), (ii) central China (between Yellow River and Yangtze River) and (iii) southern China (to the south of Yangtze River, and Xinjiang). As shown in Table 2, the highest density of the Beijing strains was observed in the northern and central part of China (85.1% and 83.9%, respectively), and the lowest was observed in the southern China (61.6%). The difference was strongly significant (P<0.001). A similar trend of geographical distribution of the Beijing strains was seen in all three age groups, children, adolescents and adults. However, the statistically significant differences among northern, central and southern China were only found for adolescents and adults (P<0.001 and 0.025, respectively), but not children (P = 0.708). In addition, in southern China, the prevalence of the Beijing family strains in children was relatively higher than that in adolescents and adults (77.8% vs. 60.4% and 59.5%).

**Table 2:** Geographical distribution of Beijing family strains in different age groups.

	Northern		Central		Southern		P value
	Beijing, n(%)	non-Beijing, n(%)	Beijing, n(%)	non-Beijing, n(%)	Beijing, n(%)	non-Beijing, n(%)	
Children group (n = 90)	55 (87.3)	8 (12.7)	15 (83.3)	3 (16.7)	7 (77.8)	2 (22.2)	0.708
Adolescent group (n = 159)	70 (89.7)	8 (10.3)	30 (90.9)	3 (9.1)	29 (60.4)	19 (39.6)	<0.001
Adult group (n = 191)	86 (80.4)	21 (19.6)	33 (78.6)	9 (21.4)	25 (59.5)	17 (40.5)	0.025
Total (n = 440)	211 (85.1)	37 (14.9)	78 (83.9)	15 (16.1)	61 (61.6)	38 (38.4)	<0.001

**Note:** Northern China: Beijing, Gansu, Hebei, Heilongjiang, Jilin, Liaoning, Inner Mongolia, Qinghai, Shanxi, Tianjin, Xizang (Tibet); Central China: Anhui, He’nan, Hubei, Jiangsu, Shangdong, Shanxi, Sichuan, Chongqing; Southern China: Fujian, Guangxi, Guizhou, Hu’nan, Jiangxi, Xinjiang.

### Distribution of the Beijing Family Strains in New and Previously Treated Cases

Table 3 shows the distribution of the Beijing family strains in new and previously treated cases in three age groups. Among 308 new cases of the total sample, the prevalence rate of the Beijing family was almost the same among newly-diagnosed (79.9%, 246/308) and previously treated cases (78.8%, 104/132). After stratification by age, Beijing strains were found to be much more prevalent among new cases in children (91.0% vs. 69.6% in the previously treated cases, P = 0.029). In contrast, the proportion of the Beijing family strains was similar between new and previously treated cases in the adolescents and adults (Table 3).

**Table 3:** Distribution of Beijing genotype strains in new cases and previously treated cases in different age groups.

	Children		P value	Adolescents		P value	Adults		P value	Total		P value
	Beijing	non-Beijing		Beijing	non-Beijing		Beijing	non-Beijing		Beijing	non-Beijing	
	N (%)	N (%)		N (%)	N (%)		N (%)	N (%)		N (%)	N (%)	
New cases	61 (91.0)	6 (9.0)	0.029	87 (78.4)	24 (21.6)	0.195	98 (75.4)	32 (24.6)	1.000	246 (79.9)	62 (20.1)	0.897
Previouslytreated cases	16 (69.6)	7 (30.4)		42 (87.5)	6 (12.5)		46 (75.4)	15 (24.6)		104 (78.8)	28 (21.2)	

### Comparison of the Beijing Genotype Strains with Drug Resistance

The relationship between the Beijing genotype strains and resistance to anti-TB drugs was analyzed (Table 4). The rates of resistance to the first-line drugs and MDR in the Beijing family strains were higher than those in the non-Beijing strains in the total collection while this difference was statistically significant for EMB resistance (P = 0.009). After stratification by age, the drug resistance rates (all tested drugs and MDR) in the Beijing strains were still higher than those in the non-Beijing strains in all three groups, with the exception of RIF and INH resistance in adolescents (34.0% vs. 38.3% for RIF, 43.8% vs. 46.8% for INH in the Beijing and non-Beijing strains, respectively). In addition, Beijing family strains were also found to be associated with INH (P = 0.028) and EMB (P = 0.027) resistance in adolescent group.

**Table 4:** Drug resistance properties of the Beijing family strains and strains of other genotypes.

	Children		P value	Adolescents		P value	Adults		P value	Total		P value
	Beijing	non-Beijing		Beijing	non-Beijing		Beijing	non-Beijing		Beijing	non-Beijing	
	N (%)	N (%)		N (%)	N (%)		N (%)	N (%)		N (%)	N (%)	
RIF	28 (36.4)	2 (15.4)	0.244	34 (26.4)	3 (10.0)	0.091	49 (34.0)	18 (38.3)	0.602	111 (31.7)	23 (25.6)	0.305
INH	26 (33.8)	3 (23.1)	0.658	44 (34.1)	4 (13.3)	0.028	63 (43.8)	22 (46.8)	0.738	133 (38.0)	29 (32.2)	0.329
STR	32 (41.6)	4 (30.8)	0.551	39 (30.2)	9 (30.0)	1	64 (44.4)	19 (40.4)	0.735	135 (38.6)	32 (35.6)	0.628
EMB	17 (22.1)	2 (15.4)	0.857	34 (26.4)	2 (6.7)	0.027	34 (23.6)	6 (12.8)	0.148	85 (24.3)	10 (11.1)	0.009
MDR	20 (26.0)	2 (15.4)	0.636	32 (24.8)	3 (10.0)	0.09	45 (31.3)	14 (29.8)	0.859	97 (27.7)	19 (21.1)	0.229

The relationship between Beijing genotype strains and first-line anti-TB drug resistance in new cases and previously treated cases was further analyzed, as shown in Table 5. The distribution of drug resistance between Beijing and non-Beijing strains among new cases and previously treated cases in the total collection was similar, with an exception for EMB resistance in previously treated cases (47.1% in Beijing strains vs. 17.9% in non-Beijing strains, P = 0.009). When the patients were divided into three age groups, no significant difference was noticed in adolescents and adults. However, in the previously treated cases in the children group, the resistance rate to all four first-line drugs and MDR was higher in Beijing genotype strains compared to strains of other genotypes (81.3% vs. 14.3% for RIF, 75.0% vs. 28.6% for INH, 81.3% vs. 14.3% for SM, 50.0% vs. 14.3% for EMB, 75.0% vs. 14.3% for MDR), and the difference was significant for RIF, SM and MDR (P = 0.01, 0.01 and 0.025, respectively).

**Table 5:** Drug resistance properties of the Beijing family strains and strains of other genotypes in subgroups of new and previously treated cases.

		Children		P value	Adolescent		P value	Adult		P value	Total		P value
		Beijing	non-Beijing		Beijing	non-Beijing		Beijing	non-Beijing		Beijing	non-Beijing	
		N (%)	N (%)		N (%)	N (%)		N (%)	N (%)		N (%)	N (%)	
New cases	RIF	15 (24.6)	1 (16.7)	1.000	11 (12.6)	0 (0)	0.147	21 (21.4)	10 (31.3)	0.339	47 (19.1)	11 (17.7)	0.858
	INH	14 (23.0)	1 (16.7)	1.000	20 (23.0)	1 (4.2)	0.073	32 (32.7)	10 (31.3)	1	66 (26.8)	12 (19.4)	0.256
	STR	19 (31.1)	3 (50.0)	0.629	18 (20.7)	6 (25.0)	0.780	37 (37.8)	9 (28.1)	0.397	74 (30.1)	18 (29.0)	0.879
	EMB	9 (14.8)	1 (16.7)	1.000	14 (16.1)	1 (4.2)	0.240	13 (13.3)	3 (9.4)	0.786	36 (14.6)	5 (8.1)	0.212
	MDR	8 (13.1)	1 (16.7)	1.000	10 (11.5)	0 (0)	0.181	20 (20.4)	7 (21.9)	1	38 (15.4)	8 (12.9)	0.694
Previously treated cases	RIF	13 (81.3)	1 (14.3)	0.010	23 (54.8)	3 (50.0)	1.000	28 (60.9)	8 (53.3)	0.764	64 (61.5)	12 (42.9)	0.088
	INH	12 (75.0)	2 (28.6)	0.102	24 (57.1)	3 (50.0)	1.000	31 (67.4)	12 (80.0)	0.546	67 (64.4)	17 (60.7)	0.825
	STR	13 (81.3)	1 (14.3)	0.010	21 (50.0)	3 (50.0)	1.000	27 (58.7)	10 (66.7)	0.763	61 (58.7)	14 (50.0)	0.520
	EMB	8 (50.0)	1 (14.3)	0.250	20 (47.6)	1 (16.7)	0.322	21 (45.7)	3 (20.0)	0.127	49 (47.1)	5 (17.9)	0.009
	MDR	12 (75.0)	1 (14.3)	0.025	22 (52.4)	3 (50.0)	1.000	25 (54.3)	7 (46.7)	0.767	59 (56.7)	11 (39.3)	0.135

## Discussion

The present study aimed to investigate the genetic diversity and drug resistance of *M. tuberculosis* strains isolated from children TB patients and compared with adolescent and adult TB patients coming from different regions across China. Although these strains might be not representative of all strains present in the country, they provide a preliminary insight into the population structure of *M. tuberculosis* circulating in these specific groups, especially in view of the total lack of this kind of data on Chinese pediatric TB population. Our study found that the Beijing family strains were prevalent in the total collection (79.5%). This result is similar to the two previous large-scale studies conducted in China [9], [10]. Dong et al. analyzed 2,346 *M. tuberculosis* isolates from 13 provinces and showed that Beijing family isolates were prevalent all over the country (74.08%) [9], while Guo et al. reported that 77.8% of 158 strains isolated in five provinces belonged to the Beijing genotype [10]. However, this rate was much lower (62.2%) in a recent study of 4017 isolates from the National Drug Resistance Base-Line Surveillance of Tuberculosis [11]. The explanation of the difference may be that the strains used in the national survey were mainly from the southern China (61.3%), where the Beijing strains showed a lower percentage (discussed below).

Here, the prevalence of the Beijing strains varied in different age groups, ranging from 85.56% in children, 81.13% in adolescents, to 75.39% in adults. Further comparison of the distribution of the Beijing strains between new cases and previously treated cases demonstrated that Beijing strains were significantly associated with new cases in children than in previously treated patients. It is known that the Beijing family strains are worldwide spread clones, some of which represent epidemic clones in some areas [12]. This particular genotype was for the first time identified in strains isolated in the Beijing area in China and thus named accordingly [13]. It was speculated that long-term *Mycobacterium bovis* BCG vaccination may be one of the selective forces implicated in the successful spread of the Beijing genotype at least in East Asia, although the association remains inconclusive [14]–[17]. Our results provide some evidence in support of the hypothesis that Beijing genotype may possess mechanisms to circumvent a BCG-induced immunity. Firstly, BCG was included in the planned vaccination program in China since mid-1970s, and the vaccination rates had already exceeded 85% after 1990. Although the BCG vaccination information was not available in our study, we can assume that the vaccination rate in children and adolescents is higher than in adults. Therefore, the Beijing family strains might be more prevalent among younger population. Secondly, our data showed that Beijing strains were significantly associated with new cases than in previously treated patients in children. The selection of strains in the previously treated cases mainly comes from the use of anti-TB drugs. However, the newly diagnosed patients did not receive any or very short (less than 1 month) drug therapy. In this sense, the significantly higher proportion of Beijing strains in new cases of children might be explained by other factors, such as higher transmission ability under the pressure of BCG vaccination.

Analysis of the geographical distribution of the Beijing family strains showed similar general trends in all three age groups: the prevalence rate of the Beijing genotype was higher in northern and central China, and lower in southern China. This result is in agreement with a previous study showing that the prevalence of the Beijing genotype exhibited geographical variation from 54.50% to 92.59% across China whereas the highest prevalence was found in northern China, followed by central and southern China [9]. Another study focused on genotypic characteristics of childhood tuberculosis in Chongqing, southern China, found that Beijing lineages accounted for 64.8% of the all strains [18]. The proportion is much lower than in our study, but is in concordance with the previous surveillance in the surrounding areas of Chongqing, such as Sichuan province (57.89%) and Hunan province (66.00%) [9]. Thus a difference between our country-wide study and Chongqing pediatric study may be explained by geographical variation. Pang et al. suggested that the geographical distribution difference of the Beijing genotype strains in China might be caused by the different economic status and climate condition [11]. In addition, compared with adolescents and adults, the prevalence of the Beijing family strains in southern China was relatively high in children. This may be due to the small size of the isolates from south China in children, and the further study including larger samples should be undertaken.

It was reported that Beijing family strains were associated with drug resistance, which might drive the spread of this particular genotype. However, the relationship between the Beijing family strains and drug resistance varied in different studies [11], [19]–[21]. A comprehensive review on the association of drug resistance with Beijing strains revealed that Beijing genotype was significantly associated with resistance in Denmark (RIF and EMB), Finland (RIF and STR), the Netherlands (STR), and Russia (all first-line drugs and MDR), while no association was found in most of the Asian studies [19]. Recently, a study analyzing 1,375 *M. tuberculosis* strains from six provinces in China revealed that Beijing strains were not associated with RIF, INH and MDR resistance [20], which was consistent with the above mentioned review [19] However, Pang and his colleagues using strains from national survey revealed that Beijing family strains exhibited a greater association with resistance to RIF, INH, EMB, OFL and MDR than other *M. tuberculosis* spoligotypes [11]. Recently, a meta-analysis on the correlation between the Beijing family strains and drug resistance also showed that in China, there was an association between Beijing genotype and resistance to RIF, EMB and MDR [21]. In our study, EMB resistance was significantly associated with the Beijing family strains in total samples, and INH and EMB resistance rates were higher in the Beijing genotype strains among adolescents. Further comparison among new cases and previously treated cases also found a strong association between EMB resistance and Beijing strains in previously treated cases in total samples, as well as resistance to RIF, STR, MDR and Beijing strains in previously treated cases of children. Our results are in agreement with the above studies [11], [21]. The differences between studies conducted in China may be due to the source of isolates. The Chinese data included in the comprehensive review were from three single studies (Shanghai, Henan and Hong Kong) [19]. Yang et al. enrolled isolates from six field sites located in six different provinces of China [20]. In contrast, the strains of Pang et al. were from nation-wide survey (31 provinces) [11], and our collection was also geographically representative (25 provinces). Finally, the meta-analysis explored publications that dealt with various sources of isolates [21]. Therefore, the results of the latter two studies [11], [21] and this study may better represent a real situation.

## Conclusion

In conclusion, this study for the first time analyzed the genetic diversity of *M. tuberculosis* from children in China at the country-wide level; its results were further compared to those obtained on the isolates from adolescents and adults. As a result, the prevalence of Beijing genotype strains was higher in northern and central China, and the lowest in southern China. The genetic characteristics of *M. tuberculosis* isolates were found similar in all three age groups, in particular marked with overall predominance of the Beijing family. The Beijing strains were significantly associated with RIF, STR and MDR resistance in previously treated children.
